# Hybrid Impulsive Control for Closed Quantum Systems

**DOI:** 10.1155/2013/545091

**Published:** 2013-05-28

**Authors:** Shouwei Zhao, Jitao Sun, Hai Lin

**Affiliations:** ^1^College of Fundamental Studies, Shanghai University of Engineering Science, Shanghai 201620, China; ^2^Department of Mathematics, Tongji University, Shanghai 200092, China; ^3^Department of Electrical Engineering, University of Notre Dame, Notre Dame, IN 46556, USA

## Abstract

The state transfer problem of a class of nonideal quantum systems is investigated. It is known that traditional Lyapunov methods may fail to guarantee convergence for the nonideal case. Hence, a hybrid impulsive control is proposed to accomplish a more accurate convergence. In particular, the largest invariant sets are explicitly characterized, and the convergence of quantum impulsive control systems is analyzed accordingly. Numerical simulation is also presented to demonstrate the improvement of the control performance.

## 1. Introduction

One of major concerns in quantum control is how to steer quantum states to a desired target state precisely and efficiently. A solution to this quantum state transfer problem will help us to advance some promising applications such as quantum computation and quantum chemistry. The main difficulty in quantum control is due to the limitations on the application of observation and feedback in quantum systems. Open-loop control has therefore been a commonly adopted approach in quantum control, where recorded control signals obtained from numerical simulations are implemented to real quantum systems. Among existing open-loop control design methods, the Lyapunov method could be the most popular one and has been tested in real applications [1–6]. Despite great advances have been made in Lyapunov methods, they may fail to achieve the control goal if the internal Hamiltonian is *not strong regular* [7]. This nonideal case means that distances between the eigenvalues of the internal Hamiltonian are not distinct. It is worth pointing out that this nonideal case does exist in many practical quantum systems such as coupled spin systems and harmonic oscillator systems [3, 4].

In particular, this paper will study the state transfer for closed quantum systems modeled as the following Schrödinger equation:

(1) $$\begin{array}{c} i|\dot{\psi }\left(t\right)\rangle =H_{0}|\psi \left(t\right)\rangle , \end{array}$$

where *H*_0_ is the internal Hamiltonian. For quantum systems, the control is implemented to the system through electromagnetic fields. Our basic problem is to transfer a quantum state from an initial state to a desired target state. The difficulty for the Lyapunov control of nonideal quantum system mainly comes from the fact that the system could be driven to undesired limit points in the invariant set. There exist a few results in the recent literature handling such a nonideal case. In [7], the complete controllability of the quantum systems with twofold degeneracy was investigated, and the basic idea is to apply a weak constant field to eliminate the degeneracy. In [4], the implicit Lyapunov method was used to deal with such a nonideal case. However, it is difficult to characterize invariant sets which are critical for the following convergence analysis. Therefore, we propose a new hybrid impulsive control strategy for closed quantum systems under the nonideal case. Nowadays, the impulsive control has proved to be an effective method to accomplish good performance for classical systems [8–12]. This motivates us to apply such a control idea to quantum systems.

The basic idea of the hybrid impulsive control is to divide the control into a piecewise continuous open-loop coherent control *u*_1_(*t*) and an impulsive control *u*_2_. *u*_1_(*t*) design is similar to the traditional Lyapunov control which drives states to invariant sets. Specifically, the system under the piecewise continuous control can be described by

(2) $$\begin{array}{c} i|\dot{\psi }\left(t\right)\rangle =\left[H_{0}+\sum_{l=1}^{r}H_{l}u_{1l}\left(t\right)\right]|\psi \left(t\right)\rangle ,\;t\neq t_{k}, \end{array}$$

where *H*_*l*_ is the control Hamiltonian and *u*_1*l*_(*t*) is real-valued control function (*l* ∈ *J* = {1,2,…, *r*}). The continuous-time coherent control *u*_1_(*t*) is implemented through the control Hamiltonians *H*_*l*_ when *t* ≠ *t*_*k*_. Due to the nonideal quantum system, this control cannot guarantee the convergence to the desired target state. After a certain instant *t*_*k*_, the controlled state would sufficiently approach an undesired limit point. Hence, the control could fail to drive the system state. Then we need to “kick” the state out of undesired limit points and drive it re-converge to new, hopefully desired, state. At *t* = *t*_*k*_, the piecewise continuous control *u*_1_(*t*) is switched off, and the “kicking” effect is accomplished by the impulsive control *u*_2_(*t*), by which the controlled system at the instant *t*_*k*_ becomes

(3) $$\begin{array}{c} i|\dot{\psi }\left(t\right)\rangle =\left[H_{0}+{\overset{-}{B}}_{k}\delta \left(s-t_{k}\right)\right]|\psi \left(t\right)\rangle ,\;t\neq t_{k}, \end{array}$$

where the introduced Hermitian operator ${\bar{B}}_{k}$ is the impulsive control Hamiltonian to be designed, *δ*(·) is the Dirac impulse function with *u*_2_(*t*) = 0, *t* ≠ *t*_*k*_, and *t*_*k*_ is the impulsive instant at which the impulsive control is implemented, satisfying lim⁡_*k*→*∞*_ *t*_*k*_ = *∞*. With the impulsive control *u*_2_(*t*), the state satisfies $|\psi (t_{k}+h)\rangle =e^{-i\int_{t_{k}}^{t_{k}+h}[H_{0}+{\overset{-}{B}}_{k}\delta (s-t_{k})]ds}|\psi (t_{k})\rangle$, for sufficiently small *h* > 0. As *h* → 0^+^, we have $\lim_{h\to 0^{+}}|\psi \left(t_{k}+h\right)\rangle =|\psi \left(t_{k}^{+}\right)\rangle =e^{-i{\bar{B}}_{k}}|\psi \left(t_{k}\right)\rangle$ and define |*ψ*(*t*_*k*_)〉 = |*ψ*(*t*_*k*_^−^)〉 = lim⁡_*h*→0^+^_ | *ψ*(*t*_*k*_ − *h*)〉. In the control process, in order to keep the coherence of the controlled state, the proposed control strategy is the open-loop coherent control.

In practical implementations, the *δ* function in the impulsive control can be substituted with pulses of finite duration if the duration is sufficiently short compared with the time scale of quantum systems [13]. In recent years, the impulsive control idea has been used in the control of open quantum systems to suppress decoherence, for example, bang-bang pulses [13–18], and the minimal time control of spin systems [19, 20]. It is realized by a sequence of unitary operations, characteristic of instantaneous pulses. The feasibility of this impulsive control was supported by physical experiments; see [14–17] and the references therein. The so called hard pulses in NMR are analogous to this picture.

With this understanding, this paper will focus on the development of hybrid impulsive control design itself to achieve more accurate convergence under the nonideal cases. Moreover, the invariant set of the controlled system is characterized explicitly, which is shown to be strictly smaller than that obtained using classical Lyapunov methods. The convergence analysis is then obtained via an extending LaSalle invariance principle for impulsive systems in [21]. Simulation studies show improved control performance.

The rest of this paper is organized as follows. In Section 2, we design the hybrid impulsive control for nonideal systems by the Lyapunov function based on the state distance. Properties of the controlled system are discussed, and the convergence is analyzed by explicit characterization of the LaSalle invariant set. In Section 3, using the Lyapunov function based on the state error, the hybrid impulsive control of quantum systems is investigated.  Section 4 includes numerical simulation to demonstrate the effectiveness and advantage of the proposed methods. Finally, some conclusions are drawn in Section 5.

## 2. Hybrid Impulsive Control Based on the State Distance

 In practice, we do not have much freedom to choose the control Hamiltonian due to the structure limitations of the control fields [3, 4]. The impulsive control Hamiltonian cannot be chosen arbitrarily to achieve the state transfer instantaneously. Thus in this paper, *H*_*l*_ is fixed and assumed to be known beforehand. Denote $B_{k}=e^{-i{\bar{B}}_{k}}$, which is unitary. With the hybrid impulsive control fields *u*(*t*), system (1) becomes a *closed quantum impulsive control system *as follows:

(4) $$\begin{array}{c} i|\dot{\psi }\left(t\right)\rangle =\left[H_{0}+\sum_{l=1}^{r}H_{l}u_{1l}\left(t\right)\right]|\psi \left(t\right)\rangle ,\;t\neq t_{k},\\ |\psi \left(t_{k}^{+}\right)\rangle =B_{k}|\psi \left(t_{k}\right)\rangle . \end{array}$$

In the following, we denote *H* = *H*_0_ + ∑_*l*=1_^*r*^ *H*_*l*_ *u*_1*l*_(*t*).

### 2.1. Hybrid Impulsive Control Design and Dynamical Properties of Controlled Systems

 In quantum control, the goal state |*ψ*_*f*_〉 is usually chosen to be an eigenstate of *H*_0_, that is, *H*_0_|*ψ*_*f*_〉 = *λ*_*f*_|*ψ*_*f*_〉. We select a Lyapunov function based on the Hilbert-Schmidt distance between |*ψ*〉 and |*ψ*_*f*_〉, that is, *V*_1_(*ψ*(*t*)) = *V*_1_(*t*) = (1/2)(1 − |〈*ψ*_*f*_ | *ψ*〉|^2^). When *t* ≠ *t*_*k*_, the time derivative of *V*_1_ is given by

(5) $$\begin{array}{c} {\dot{V}}_{1}\left(t\right)=-\sum_{l=1}^{r}u_{1l}ℑ\left[\langle \psi |\psi_{f}\rangle \langle \psi_{f}|H_{l}|\psi \rangle \right], \end{array}$$

where *ℑ*(·) and *ℜ*(·) denote the imaginary part and real part of a complex number, respectively. When *t* = *t*_*k*_, the Dini derivative of *V*_1_ is given by *D* ^−^ *V*_1_(*t*_*k*_)≜lim⁡_*h*→0^+^_  ((*V*_1_(*t*_*k*_) − *V*_1_(*t*_*k*_ − *h*))/*h*). The difference of *V*_1_ is described as Δ*V*_1_(*t*_*k*_) = *V*_1_(*t*_*k*_^+^) − *V*_1_(*t*_*k*_).

In order that the designed control can work in the case of the initial state being orthogonal to the goal state, we rewrite (5) as ${\dot{V}}_{1}(t)=-\sum_{l=1}^{r}u_{1l}|\langle \psi |\psi_{f}\rangle |ℑ[e^{i∠\langle \psi |\psi_{f}\rangle }\langle \psi_{f}|H_{l}|\psi \rangle ]$. We need to design the control law such that ${\dot{V}}_{1}(t)\leq 0$, *t* ≠ *t*_*k*_ and Δ*V*_1_(*t*_*k*_) ≤ 0. Choose the piecewise continuous control law as follows:

(6) $$\begin{array}{c} u_{1l}\left(t\right)=K_{l}f_{l}\left(ℑ\left[e^{i∠\langle \psi |\psi_{f}\rangle }\langle \psi_{f}|H_{l}|\psi \rangle \right]\right),\;l\in J,\;t\neq t_{k}, \end{array}$$

where *K*_*l*_ > 0 is the control gain and the function *f*_*l*_(·) passes through the origin of plane *x*_*l*_ − *y*_*l*_ monotonically satisfying *f*_*l*_(*x*_*l*_)*x*_*l*_ ≥ 0 with *x*_*l*_ = *ℑ*[〈*ψ*_*f*_ | *H*_*l*_ | *ψ*〉]. To avoid the confusion, we define *∠*〈*ψ* | *ψ*_*f*_〉 = 0° if 〈*ψ* | *ψ*_*f*_〉 = 0. In addition, the impulsive control matrix *B*_*k*_ should be chosen to satisfy Δ*V*_1_(*t*_*k*_) ≤ 0; that is,

(7) $$\begin{array}{c} B_{k}^{*}\rho_{f}B_{k}-\rho_{f}\geq 0, \end{array}$$

where *ρ*_*f*_ = |*ψ*_*f*_〉〈*ψ*_*f*_| is the density matrix of target state |*ψ*_*f*_〉. Inequality (7) holds at least for the unitary matrix *B*_*k*_ which commutes with *ρ*_*f*_. The control law satisfying (6) and (7) is the designed control law. In the following, the properties of system (4) will be studied to show that *u*(*t*) can make the system leave the initial state even if |*ψ*(0)〉 is an eigenstate of *H*_0_.

**Lemma 1**

For control law (6) and (7), if the initial state is an eigenstate of *H*_0_ with *H*_0_|*ψ*(0)〉 = *λ*_0_|*ψ*(0)〉 and 〈*ψ*(0) | *ψ*_*f*_〉 = 0, then the following conclusions hold:

- if there exists *l* ∈ *J* such that *ℑ*〈*ψ*_*f*_ | *H*_*l*_ | *ψ*(0)〉≠0, then 〈*ψ*(*t*) | *ψ*_*f*_〉 ≠ 0  (*t* > 0);
- if *ℑ*〈*ψ*_*f*_ | *H*_*l*_ | *ψ*(0)〉 = 0, for all *l* ∈ *J*, and there exists *l* ∈ *J* such that *ℜ*〈*ψ*_*f*_ | *H*_*l*_ | *ψ*(0)〉≠0 and *λ*_0_ ≠ 0, then 〈*ψ*(*t*) | *ψ*_*f*_〉≠0, *t* > *t*′ > 0, where *t*′ is sufficiently small; otherwise, the designed control fields cannot achieve the state steering of the closed-loop system.

**Proof**

(i) For a sufficiently small *dt*, as *t* ≠ *t*_*k*_, we have $i|\dot{\psi }\left(0\right)\rangle =i\;\lim_{dt\to 0\;}\;[|\psi (dt)\rangle \;-|\psi (0)\rangle ]/dt=H|\psi (0)\rangle$; that is, as *dt* → 0, |*ψ*(*dt*)〉 = (*I* − *iH* *dt*) | *ψ*(0)〉. Since 〈*ψ*(0) | *ψ*_*f*_〉 = 0, the inequality 〈*ψ*_*f*_ | *ψ*(*dt*)〉≠0 is equivalent to ∑_*l*=1_^*r*^ *u*_1*l*_〈*ψ*_*f*_ | *H*_*l*_ | *ψ*(0)〉 = ∑_*l*=1_^*r*^ *u*_1*l*_(*iℑ*〈*ψ*_*f*_ | *H*_*l*_ | *ψ*(0)〉+*ℜ*〈*ψ*_*f*_ | *H*_*l*_ | *ψ*(0)〉) ≠ 0. By (6), we have *u*_1*l*_ *ℑ*〈*ψ*_*f*_ | *H*_*l*_ | *ψ*(0)〉≥0, for all *l* ∈ *J*. It follows from the assumption in case (i) that there exists *l* ∈ *J* such that *u*_1*l*_ *ℑ*〈*ψ*_*f*_ | *H*_*l*_ | *ψ*(0)〉>0, and consequently, 〈*ψ*_*f*_ | *ψ*(*dt*)〉≠0. Since ${\dot{V}}_{1}\leq 0$, we obtain that 〈*ψ*(*t*) | *ψ*_*f*_〉≠0, *t* ∈ (0, *t*_1_). By (7) we have |〈*ψ*_*f*_ | *ψ*(*t*_1_^+^)〉|≥|〈*ψ*_*f*_ | *ψ*(*t*_1_)〉| = |〈*ψ*_*f*_ | *ψ*(*t*_1_^−^)〉|>0. Hence, we obtain that 〈*ψ*(*t*) | *ψ*_*f*_〉≠0, (*t* > 0).

(ii) Initially, the system evolves freely because *u*_1*l*_(0) = 0, *l* ∈ *J*. For a sufficiently small t* < *t*_1_, one can obtain that |*ψ*(t*)〉 = *e* ^−*iH*_0_t*^ | *ψ*(0)〉 = *e* ^−*iλ*_0_t*^ | *ψ*(0)〉. Moreover, as *dt* → 0, we have 〈*ψ*_*f*_ | *ψ*(t* + *dt*)〉 = 〈*ψ*_*f*_ | (*I* − *iH* *dt*) | *ψ*(t*)〉 = *dt*[−*i*  cos⁡(*λ*_0_ t*) − sin(*λ*_0_ t*)]∑_*l*=1_^*r*^〈*ψ*_*f*_ | *H*_*l*_ *u*_1*l*_(t*) | *ψ*(0)〉. Noticing that there exists *l* ∈ *J* such that 〈*ψ*_*f*_ | *H*_*l*_ | *ψ*(0)〉≠0, we have *u*_1*l*_(t*) = *K*_*l*_ *f*_*l*_[*ℑ*(*e* ^−*iλ*_0_t*^〈*ψ*_*f*_ | *H*_*l*_ | *ψ*(0)〉)] = *K*_*l*_ *f*_*l*_[−sin(*λ*_0_ t*)〈*ψ*_*f*_ | *H*_*l*_ | *ψ*(0)〉] ≠ 0. Similar to the discussion in case (i), we obtain that 〈*ψ*_*f*_ | *ψ*(t* + *dt*)〉≠0, and then 〈*ψ*(*t*) | *ψ*_*f*_〉≠0, *t* > t*. This completes the proof.

For the characterization of invariant sets, properties of the states such that ${\dot{V}}_{1}=0$ are studied. Since the proof is similar to that of Proposition 4 in [1], we omit it here.

**Lemma 2**

If 〈*ψ*_0_ | *ψ*_*f*_〉 = 0 and the conditions (i) or (ii) in Lemma 1 hold, the following conclusions are equivalent:

- ${\dot{V}}_{1}(t)=0$, *t* ≠ *t*_*k*_,
- $i|\dot{\psi }\left(t\right)\rangle =H_{0}|\psi \left(t\right)\rangle$, *t* ≠ *t*_*k*_,
- there exists *λ*_*l*_ ∈ ℝ such that 〈*ψ*_*f*_|(*λ*_*l*_ *I* − *H*_*l*_)|*ψ*(*t*)〉 = 0, *t* ≠ *t*_*k*_, *l* ∈ *J*.

Lemma 2 only characterizes the states guaranteeing that ${\dot{V}}_{1}=0$ at specific instants. We need to characterize the states from which the system trajectories stay in the set ${\dot{V}}_{1}=0$ and Δ*V*_1_ = 0. We first present the extensive LaSalle invariance principle for impulsive systems in [21].

**Lemma 3**

Consider the following differential impulsive system on an open set *𝒟*:

(8) x˙(t)=fc(x(t)), x(0)=x0,  t≠tk,Δx(t)=fd(x(t)), t=tk.

If there exists a continuous function *V* such that *V*′(*x*)*f*_*c*_(*x*) ≤ 0, *t* ≠ *t*_*k*_ and Δ*V*(*t*_*k*_) ≤ 0, then *x*(*t*) → *ℳ* as *t* → *∞*, where *ℳ* is the largest invariant set contained in *𝔼*≜{*x* : *V*′(*x*)*f*_*c*_(*x*) = 0}∩{*x* : Δ*V*(*x*) = 0}.

### 2.2. Convergence Analysis

 The following theorem presents the characterization of the invariant set for the nonideal systems under the hybrid impulsive control, by which the invariant set is smaller compared with that obtained by the conventional Lyapunov method. In the following, the unitary matrix *B*_*k*_ is designed such that it commutes with *H*_0_, *k* ∈ *ℤ* ^+^.

**Theorem 4**

Consider system (4) with the hybrid impulsive control satisfying (6) and (7). The largest invariant set is given by *G* = *𝕊* ^2*n*−1^⋂*E*_1_⋂*E*_2_ with *E*_1_ = {|*ψ*〉 : |*ψ*〉 ∈ *M*_*k*_^*l*^, for  all  *l* ∈ *J*, *k* = 1,2,…}, *E*_2_ = {|*ψ*〉 : |*ψ*〉 ∈ *N*_*k*_, *k* = 1,2,…}, and

(9) M1l:={|ψ〉:ℑ(〈ψ|ψf〉〈ψf|Xlsl|ψ〉)=0,      sl=1,2,…,ml},Mkl  ≔{|ψ〉:ℑ(〈ψ|∏j=1k−1Bj∗|ψf〉〈ψf|Xlsl∏j=k−11Bj|ψ〉)      =0,sl=1,2,…,ml}, k≥2,Nk  :={|ψ〉:|〈ψ|∏j=1k−1Bj|ψf〉|2−|〈ψ|ψf〉|2=0}, k≥1,

where *X*_*l*_^1^, *X*_*l*_^2^,…, *X*_*l*_^*m*_*l*_^ constitute the basis of the set {(*i*)^*s*^[*H*_0_^(*s*)^, *H*_*l*_], *s* = 0,1, 2,…}, *l* ∈ *J*. Hence, system (4) converges to *G* under the hybrid impulsive control.

**Proof**

When *t* = *t*_0_, from (6), we obtain that

(10) V˙1(t0)=0⇔|〈ψ(t0)|ψf〉| ×ℑ[ei∠〈ψ(t0)|ψf〉〈ψf|Hl|ψ(t0)〉]=0, l∈J⇔ℑ(〈ψ(t0)|ψf〉〈ψf|Hl|ψ(t0)〉)=0.

The main idea of the proof is sketched as follows. The interval [*t*_*k*−1_, *t*_*k*_] is divided into *n*_*k*_ sufficiently small intervals with duration *dt*. We apply the Taylor expansion on the system state and omit the high order terms of *dt*. By Lemma 2, the requirements ${\dot{V}}_{1}\left(t\right)=0\;(t\neq t_{k})$ and Δ*V*_1_(*t*_*k*_) = 0 for the whole system trajectory will be transformed to the conditions on the initial state. By the Taylor expansion and commutativity between *H*_0_ and *B*_*k*_, it yields that

(11) V˙1(t0+dt)=0 ⇔ℑ(〈ψ(t0+dt)|ψf〉〈ψf|Hl|ψ(t0+dt)〉)=0 ⇔ℑ(〈ψ(t0)|(I+iH0dt)|ψf〉      ×〈ψf|Hl(I−iH0dt)|ψ(t0)〉)=0 ⇔ℑ(i〈ψ(t0)|ψf〉〈ψf|[H0,Hl]|ψ(t0)〉)=0,        ⋮D−V1(t1)=0 ⇔ℑ((i)n1〈ψ(t0)|ψf〉       ×〈ψf|[H0(n1),Hl]|ψ(t0)〉)=0.

At *t* = *t*_*k*_ + *dt*, the free evolution of |*ψ*(*t*)〉 is given by

(12) |ψ(tk+dt)〉=(I−iH0dt)|ψ(tk+)〉=Bk(I−iH0dt)|ψ(tk)〉, k=1,2,….

Similar to the previous deduction, it follows from (11) and (12) that

(13) V˙1(t1+dt)=0  ⇔ℑ(〈ψ(t1+dt)|ψf〉〈ψf|Hl|ψ(t1+dt)〉)=0  ⇔ℑ((i)s〈ψ(t1+)|ψf〉       ×〈ψf|[H0(s),Hl]|ψ(t1+)〉)=0, s=0,1  ⇔ℑ((i)s〈ψ(t1−dt)|B1∗|ψf〉         ×〈ψf|[H0(s),Hl]B1|ψ(t1−dt)〉)=0,                        s=0,1,2,  ⇔ℑ((i)s〈ψ(t0)|B1∗|ψf〉       ×〈ψf|[H0(s),Hl]B1|ψ(t0)〉)=0,                 s=0,1,…,n1+1,D−V1(t2)=0 ⇔ℑ((i)s〈ψ(t0)|B1∗|ψf〉     ×〈ψf|[H0(s),Hl]B1|ψ(t0)〉)=0,             s=0,1,…,n1+n2.

Consequently, it can be obtained that

(14) D−V1(tk)=0 ⇔ℑ((i)s〈ψ(t0)|∏j=1k−1Bj∗|ψf〉       ×〈ψf|[H0(s),Hl]∏j=k−11Bj|ψ(t0)〉)=0,

where *s* = 0,1,…, ∑_*i*=1_^*k*^ *n*_*i*_. Noticing that the set {(*i*)^*s*^[*H*_0_^(*s*)^, *H*_*l*_], *s* = 0,1,…, ∑_*i*=1_^*k*^ *n*_*i*_}, *l* ∈ *J* has finite dimension, we denote its basis to be *X*_*l*_^1^, *X*_*l*_^2^,…, *X*_*l*_^*m*_*l*_^, *l* ∈ *J*. Since the division of the interval [*t*_*k*−1_, *t*_*k*_] is random, (14) can be rewritten as

(15) D−V1(tk)=0 ⇔ℑ(〈ψ(t0)|∏j=1k−1Bj∗|ψf〉       ×〈ψf|Xlsl∏j=k−11Bj|ψ(t0)〉)=0,                   sl=1,…,ml.

For convenience, the set of the states satisfying (15) is denoted as *M*_*k*_^*l*^ in (9), *l* ∈ *J*, *k* ≥ 2.

In the following, we will discuss the conditions on the initial states from which the trajectories stay in the set {|*ψ*〉 : Δ*V*_1_(*t*_*k*_) = 0, *k* = 1,2,…}:

(16) ΔV1(t1)=0 ⇔|〈ψ(t1)|B1∗|ψf〉|2−|〈ψ(t1)|ψf〉|2=0 ⇔|〈ψ(t1−dt)|B1∗|ψf〉|2  −|〈ψ(t1−dt)|ψf〉|2=0 ⇔|〈ψ(t0)|B1∗|ψf〉|2−|〈ψ(t0)|ψf〉|2=0.

Applying the similar technique, it follows that

(17) ΔV1(tk)=0⇔|〈ψ(t0)|∏j=1kBj∗|ψf〉|2   −|〈ψ(t0)|ψf〉|2=0.

We denote the set of the states guaranteeing (17) to be *N*_*k*_ in (9).

In conclusion, all the states which stay in the intersection *E*_1_∩*E*_2_ constitute the largest invariant set of system (4). By Lemma 3, we complete the proof.

It should be noticed that the basis of the set {(*i*)^*s*^[*H*_0_^(*s*)^, *H*_*l*_], *s* = 0,1, 2,…, ∑_*i*=1_^*k*^ *n*_*i*_} can be obtained in finite steps, *l* ∈ *J*. If the first *n* ^2^ elements in the set are linearly independent, then they constitute the basis. If [*H*_0_^(*s*_*l*_+1)^, *H*_*l*_] can be represented by the first *s*_*l*_ elements, it is easy to obtain that [*H*_0_^(*k*)^, *H*_*l*_] can be represented by the linear combination of *H*_*l*_, [*H*_0_, *H*_*l*_],…, [*H*_0_^(*s*_*l*_)^, *H*_*l*_], for all *k* > *s*_*l*_.

**Corollary 5**

Consider system (4) with control field (6) without the impulsive control, that is, *B*_*k*_ = *I*, *k* = 1,2,…. The largest invariant set is *E*⋂*𝕊* ^2*n*−1^, where $E=\{|\psi \rangle :|\psi \rangle \in {\bar{M}}_{k}^{l},l\in J,k=0,1,\ldots \}$, ${\bar{M}}_{k}^{l}=\{|\psi \rangle :ℑ(i^{k}\langle \psi |\psi_{f}\rangle \langle \psi_{f}|[H_{0}^{\left(k\right)},H_{l}]|\psi \rangle )=0\}$, *k* ≥ 0, and *l* ∈ *J*.

**Remark 6**

If *H*_0_ is strong regular, then the result in Corollary 5 reduces to Theorem 2 in [1]. For the nonideal case, it is clear that *G* ⊂ *E*. From the viewpoint of physics, this implies that the proposed hybrid impulsive control can achieve more accurate convergence under the nonideal case. In general, the matrix *B*_*k*_ can be chosen to guarantee that *E*_1_ and *E*_2_ contain finite sets *M*_*k*_^*l*^ and *N*_*k*_. It can be found that the invariant set *G* depends on the choice of impulsive control matrix *B*_*k*_. The optimal determination of *B*_*k*_ and impulsive instants *t*_*k*_ to minimize the invariant set are under study.

## 3. Hybrid Impulsive Control Based on the State Error

 It is known that different Lyapunov functions may have different control effects. The relations among them were studied in our previous work [4]. In this section, we consider the hybrid impulsive control of quantum systems based on the state error between the controlled state and the goal state. Let *V*_2_(*ψ*(*t*)) = *V*_2_(*t*) = (1/2)〈*ψ*(*t*) − *ψ*_*f*_ | *ψ*(*t*) − *ψ*_*f*_〉 = 1 − *ℜ*〈*ψ*_*f*_ | *ψ*(*t*)〉. Similar to the hybrid control design in Section 1, we consider the following quantum impulsive control system which is different from (4):

(18) $$\begin{array}{c} i|\dot{\psi }\left(t\right)\rangle =\left(H_{0}+u_{1}H_{1}+\omega I\right)|\psi \left(t\right)\rangle ,\;t\neq t_{k},\\ |\psi \left(t_{k}^{+}\right)\rangle =B_{k}|\psi \left(t_{k}\right)\rangle , \end{array}$$

where *ω* is a new real scalar control field. For the convenience of the computation, the introduced *ω* may be used to adjust the global phase without changing the physical quantities regarding |*ψ*〉. While in practical implementation, it is not necessary to be implemented to the system. Similar conclusion can be drawn if there exists more than one control Hamiltonian *H*_*l*_, *l* ≥ 2. The time derivative of *V*_2_ is

(19) V˙2(t)=−(λf+ω)ℑ(〈ψf|ψ〉)−ℑ(〈ψf|H1|ψ〉)u1, t≠tk.

Let *u*_0_ = *λ*_*f*_ + *ω*. We design the following control to ensure ${\dot{V}}_{2}(t)\leq 0$, *t* ≠ *t*_*k*_:

(20) λf+ω=u0=K0f0(ℑ〈ψf|ψ〉),u1=K1f1(ℑ〈ψf|H1|ψ〉),

where *K*_1_ > 0, and the function *y*_1_ = *f*_1_(·) is defined as that in (6).

**Theorem 7**

Consider system (18) with control fields (7) and (20). The largest invariant set is given by *K* = *F*_1_⋂*F*_2_⋂*𝕊* ^2*n*−1^, where *F*_1_ = {|*ψ*〉:|*ψ*〉∈*U*_*k*_, *k* = 1,2,…}, *F*_2_ = {|*ψ*〉:|*ψ*〉∈*W*_*k*_, *k* = 1,2,…}, and *U*_1_ : = {|*ψ*〉:*ℑ*(〈*ψ*_*f*_ | *X* ^*s*^ | *ψ*〉) = 0, *s* = 1,…, *m*_1_},

(21) Uk ≔{|ψ〉:ℑ(〈ψf|Xs∏j=k−11Bj|ψ〉)=0,s=1,…,m1},                               k≥2,Wk :={|ψ〉:ℜ〈ψf|ψ〉−ℜ〈ψf|∏j=1kBj|ψ〉=0}, k≥1,

where *X* ^1^, *X* ^2^,…, *X* ^*m*_1_^ are the basis of the set {*I*, (*i*)^*s*^[*H*_0_^(*s*)^, *H*_1_], *s* = 0,1, 2,…}. Therefore, system (18) converges to *K* with the hybrid impulsive control satisfying (7) and (20).

**Proof**

Let *ω* = −*λ*_*f*_. When the system satisfies ${\dot{V}}_{2}=0$, that is, *u*_1_ = 0, the evolution of system (18) becomes

(22) $$\begin{array}{c} i|\dot{\psi }\left(t\right)\rangle =\left(H_{0}-\lambda_{f}I\right)|\psi \left(t\right)\rangle ,\;t\neq t_{k},\\ |\psi \left(t_{k}^{+}\right)\rangle =B_{k}|\psi \left(t_{k}\right)\rangle . \end{array}$$

It follows from (22) that

(23) |ψ(tk−1+dt)〉  =|ψ(tk−1)〉+|ψ˙(tk−1)〉dt  =[I−i(H0−λfI)dt]|ψ(tk−1)〉.

From (20), we obtain the following relation:

(24) V˙2(t0)=0⇔ℑ(〈ψf|ψ(t0)〉)=0,ℑ(〈ψf|H1|ψ(t0)〉)=0.

Similarly, we divide the interval [*t*_*k*−1_, *t*_*k*_] into *n*_*k*_ sufficiently small intervals. From (22)–(24), we have *D* ^−^ *V*_2_(*t*_1_) = 0⇔*ℑ*(*i* ^*n*_1_^〈*ψ*_*f*_ | [*H*_0_^(*n*_1_)^, *H*_1_] | *ψ*(*t*_0_)〉) = 0. According to the similar method in the proof of Theorem 4, when *t* = *t*_*k*_, it yields that

(25) D−V2(tk)=0 ⇔ℑ〈ψf|∏j=k−11Bj|ψ(t0)〉=0,      ℑ(is〈ψf|[H0(s),H1]∏j=k−11Bj|ψ(t0)〉)=0,

where *s* = 0,…, ∑_*i*=1_^*k*^ *n*_*i*_. Denote the basis of the set {*I*, (*i*)^*s*^[*H*_0_^(*s*)^, *H*_1_], *s* = 0,1,…, ∑_*i*=1_^*k*^ *n*_*i*_} to be *X* ^1^, *X* ^2^,…, *X* ^*m*_1_^. Equation (25) can be rewritten as *D* ^−^ *V*_2_(*t*_*k*_) = 0⇔*ℑ*〈*ψ*_*f*_ | *X* ^*s*^∏_*j*=*k*−1_^1^ *B*_*j*_ | *ψ*(*t*_0_)〉 = 0, *s* = 1,2,…, *m*_1_. This equality is denoted as *U*_*k*_ in (21).

Next, we characterize the initial states from which the system trajectories stay in {|*ψ*〉:Δ*V*_2_(*t*_*k*_) = 0, *k* = 1,2,…}. From the definition of *V*_2_, we have Δ*V*_2_(*t*_1_) = *ℜ*〈*ψ*_*f*_ | *ψ*(*t*_1_^+^)〉−*ℜ*〈*ψ*_*f*_ | *ψ*(*t*_1_)〉 = *ℜ*〈*ψ*_*f*_ | *B*_1_ | *ψ*(*t*_1_)〉−*ℜ*〈*ψ*_*f*_ | *ψ*(*t*_1_)〉. The following relations can be obtained:

(26) ΔV2(t1)=0 ⇔ℜ〈ψf|ψ(t1)〉−ℜ〈ψf|B1|ψ(t1)〉=0 ⇔ℜ〈ψf|(I−B1)[I−i(H0−λfI)dt]      ×|ψ(t1−dt)〉=0  ⇔ℜ〈ψf|ψ(t0)〉−ℜ〈ψf|B1|ψ(t0)〉=0.

By similar deduction, it yields that Δ*V*_2_(*t*_*k*_) = 0⇔*ℜ*〈*ψ*_*f*_ | *ψ*(*t*_0_)〉−*ℜ*〈*ψ*_*f*_ | ∏_*j*=1_^*k*^ *B*_*j*_ | *ψ*(*t*_0_)〉 = 0, which can be denoted as *W*_*k*_ in (21).

In conclusion, all the states which remain in the intersection *F*_1_∩*F*_2_ constitute the largest invariant set of controlled system (18). By Lemma 3, the proof is completed.

Similar to the discussion in Corollary 5, Theorem 7 can be reduced to Theorem 8 in [1] if there is no impulsive control, and *H*_0_ is strong regular. We can see that our result reduces the invariant set for the nonideal case. This implies that the proposed hybrid impulsive control scheme can accomplish more accurate state transfer.

## 4. Numerical Simulation

**Example 1**

**Figure 1:**
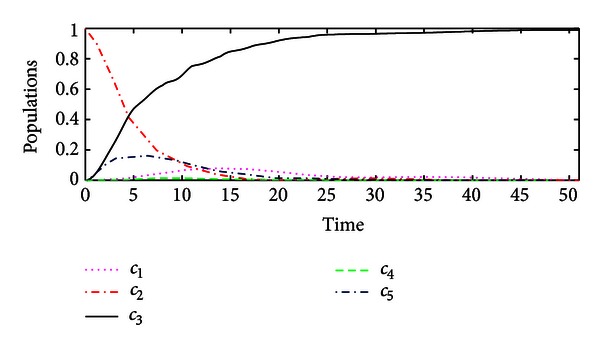
The population of the system state by the hybrid impulsive control based on the state distance.

Consider the five-level system with the internal Hamiltonian and impulsive control Hamiltonian given by *H*_0_ = diag⁡{1.0,  1.2,  1.2,  2.0,  2.15} and ${\bar{B}}_{k}=\mathrm{diag}\{0,0,-\pi ,-\pi ,0\}$, respectively. The unitary operation $B_{k}=e^{-i{\bar{B}}_{k}}=\mathrm{diag}\{1,1,\;-1,\;-1,1\}$ can be realized by performing the planar rotation on system states. It can be found that the system is a nonideal system. The control Hamiltonians are given by $H_{1}=\left(\begin{array}{ccccc} 0 & 0 & i & i & 0\\ 0 & 0 & i & 0 & i\\ -i & -i & 0 & 0 & 0\\ -i & 0 & 0 & 0 & 0\\ 0 & -i & 0 & 0 & 0 \end{array}\right)$, $H_{2}=\left(\begin{array}{ccccc} 0 & i & 0 & 0 & 0\\ -i & 0 & 0 & i & 0\\ 0 & 0 & 0 & i & i\\ 0 & -i & -i & 0 & i\\ 0 & 0 & -i & -i & 0 \end{array}\right)$. Let the target state be $|\psi_{f}\rangle ={\left[\begin{array}{ccccc} 0 & 0 & 1 & 0 & 0 \end{array}\right]}^{⊤}$, let the initial state be $|\psi_{0}\rangle ={\left[\begin{array}{ccccc} 0 & 1 & 0 & 0 & 0 \end{array}\right]}^{⊤}$, and let the controlled state be |*ψ*〉 = [*c*_1_, *c*_2_,…, *c*_5_]^*⊤*^. Take the control function to be *f*_*i*_(*x*) = *x*, *i* = 1,2. Choose the impulsive instant to be *t*_*k*_ = 3*k* − 1, *k* ∈ *ℤ* ^+^ and *K*_1_ = *K*_2_ = 0.2. Using the hybrid impulsive control based on the state distance, simple computation yields that the invariant set *G* = {|3〉} (without regard to the global phase), which implies that under the hybrid impulsive control the system converges to |*ψ*_*f*_〉. The populations of the controlled system are illustrated in Figure 1.

**Figure 2:**
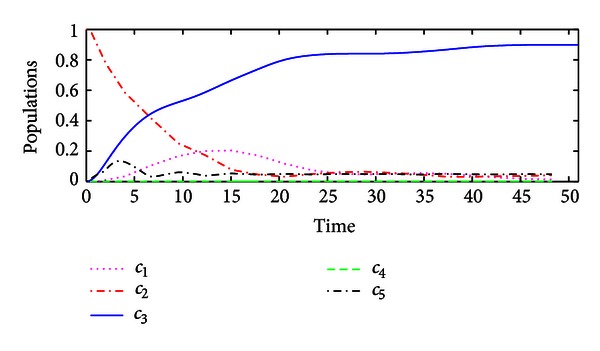
The population of the system state by the Lyapunov control without impulsive control.

Now we compare performance of the hybrid impulsive control with that of classical Lyapunov control. If the impulsive control is not applied to the system, then the hybrid impulsive control is reduced to the classical Lyapunov control, by which the performance of the controlled system is shown in Figure 2. Hence, the proposed hybrid impulsive control improves the control performance.

**Example 2**

**Figure 3:**
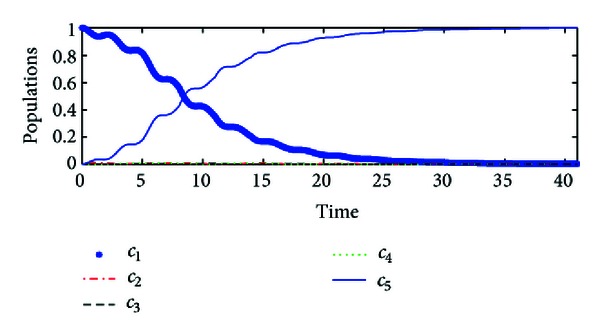
The population of the system state by the hybrid impulsive control based on the state error.

Consider the five-level system with the same internal Hamiltonian as the previous example. Let the target state and the initial state be $|\psi_{f}\rangle ={\left[\begin{array}{ccccc} 0 & 0 & 0 & 0 & 1 \end{array}\right]}^{⊤}$ and $|\psi_{0}\rangle ={\left[\begin{array}{ccccc} 1 & 0 & 0 & 0 & 0 \end{array}\right]}^{⊤}$, respectively, and the impulsive control Hamiltonian ${\bar{B}}_{k}=\mathrm{diag}\{-\pi ,0,0$,−*π*, 0}. The unitary operation is chosen as $B_{k}=e^{-i{\bar{B}}_{k}}=\mathrm{diag}\{-1,\;1,1,-1,1\}$. The system is a nonideal system. The control Hamiltonian is given by $H_{1}=\left(\begin{array}{ccccc} 0 & i & 0 & 0 & i\\ -i & 0 & 0 & i & i\\ 0 & 0 & 0 & 0 & i\\ 0 & -i & 0 & 0 & i\\ -i & -i & -i & -i & 0 \end{array}\right)$. Let the control function be *f*_1_(*x*) = *x*, and *K*_0_ = 0.1, *K*_1_ = 0.2. Choose the impulsive instant as *t*_*k*_ = 3*k* − 1, *k* ∈ *ℤ* ^+^. Using the hybrid impulsive control based on the state error, simple computation yields that the invariant set *K* = {|5〉} (without regard to the global phase), which implies that under the hybrid impulsive control the system converges to |*ψ*_*f*_〉. Simulation results are illustrated in Figure 3.

**Figure 4:**
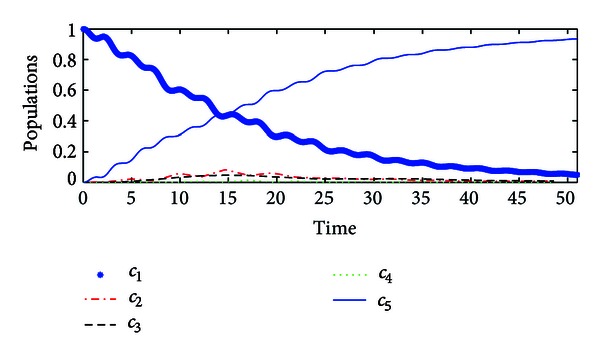
The population of the system state by the Lyapunov control without impulsive control.

**Figure 5:**
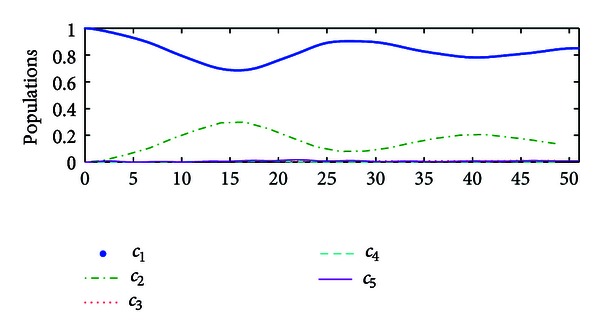
The population of the system state by the implicit Lyapunov control in [4].

When the hybrid impulsive control is reduced to the classical Lyapunov control, the trajectory of the controlled system is plotted in Figure 4. Moreover, if the implicit Lyapunov control strategy in [4] is employed with the same parameters, it fails to drive the system, as illustrated in Figure 5. Therefore, the proposed hybrid impulsive control improves the control performance.

## 5. Conclusion

 In this paper, the coherent hybrid impulsive control for closed quantum systems has been investigated for the nonideal case that *H*_0_ is not strong regular. The dynamical properties of the resulted quantum impulsive control system have been discussed to facilitate the convergence analysis. Based on two kinds of Lyapunov functions, the largest invariant sets have been characterized explicitly. Consequently, more accurate convergence of the controlled system has been achieved by the extensive LaSalle invariance principle. Compared with some existing results, the improved control performance has been shown for the nonideal case. Since the practical implementation of impulsive control has been studied in known literature, we believe that it is feasible. The optimal determination of the impulsive control Hamiltonian and impulsive instants is worth to be explored in the future work.
